# Construction and optimization of representative actual driving cycles based on the improved autoencoder

**DOI:** 10.1038/s41598-024-52865-z

**Published:** 2024-01-29

**Authors:** Zhichao Zhao, Xilei Sun, Xun Wang, Yi Wang, Jianqin Fu, Jingping Liu

**Affiliations:** 1 China Automotive Engineering Research Institute Co., Ltd., Chongqing, 401122 China; 2 New Energy Technology of CAERI Co., Ltd., Chongqing, 401122 China; 3 State Key Laboratory of Advanced Design and Manufacturing for Vehicle Body, Hunan University, Changsha, 410082 China; 4 Chongqing Research Institute of Hunan University, Chongqing, 401120 China

**Keywords:** Mechanical engineering, Scientific data, Computer science

## Abstract

In this study, much work has been performed to accurately and efficiently develop representative actual driving cycles. Electric vehicle road tests were conducted and the associated data were gathered based on the manual driving method, and the Changsha Driving Cycle Construction (CS-DCC) method was proposed to achieve systematical construction of a representative driving cycle from the original data. The results show that the refined data exhibit greater stability and a smoother pattern in contrast to the original data after noise reduction by five-scale wavelet analysis. The Gaussian Kernel Principal Component Analysis (KPCA) algorithm is chosen to reduce the dimensionality of the characteristic matrix, and the number of principal components is selected as 5 with a cumulative contribution rate of 85.99%. The average error of the characteristic parameters between the optimized drive cycle and the total data is further reduced from 13.6 to 6.1%, with a reduction ratio of 55.1%. Meanwhile, the constructed driving cycle has prominent local characteristics compared with four standard driving cycles, demonstrating the necessity of constructing an actual driving cycle that reflects localized driving patterns. The findings present a powerful application of artificial intelligence in advancing engineering technologies.

## Introduction

The driving cycle delineates the intricate correlation between the temporal progression of vehicle speed within a specific setting, which is commonly referred to as the vehicle test cycle^1,2^. As an essential fundamental technology for the automotive industry, the driving cycle plays a vital role in the realms of vehicle advancement, assessment and experimentation^3^. It is also the leading benchmark when optimizing various vehicle performance indicators^4,5^. With the growth of vehicle ownership, the gap between the actual fuel consumption and the results of regulatory certification using standard driving cycles is gradually widening^6,7^. Simultaneously, regional disparities in topography, climatic conditions, socioeconomic advancement, road topologies, and traffic patterns engender considerable variation, rendering the adoption of a uniform driving cycle impractical across diverse regions^8,9^. At present, individual nations are endeavoring to devise driving cycles tailored to the idiosyncrasies of their actual road networks during the development, testing and evaluation of automobiles^10,11^. As the most critical basic technology for the automotive industry, it is increasingly essential to develop actual driving cycles that fit the country or even a specific city^12,13^. Therefore, in order to save development costs and improve reusability, it is necessary to propose a method for facilitating the transition from original vehicle data to a final representative driving cycle^14^.

In order to construct the actual driving cycle with regional characteristics, numerous scholars have carried out extensive empirical vehicle tests and research endeavors. Amirjamshidi et al.^15^ used simulation data derived from a coordinated micro-traffic model of the Toronto waterfront to generate a representative driving cycle, which had distinct regional characteristics and high emission factors. Representative driving cycles tailored for passenger cars and motorcycles were developed to reflect the authentic driving conditions of Chennai in Ref.^16^, resulting in two driving cycles of 1448 and 1065 s, respectively. Berzi et al.^17^ obtained a driving cycle by monitoring a fleet of electric vehicles and employing pseudo-random selection of raw data, and the results demonstrated commendable regenerative braking capacity and smooth traction at low speeds of the cycle. A new driving cycle construction method based on a two-level optimization process was proposed in Ref.^18^, which produced a more representative driving cycle that was closer to the statistical data of 2.49% than the traditional Markov chain (MC) method. Cui et al.^19^ presented a novel method based on simulated annealing (SA) algorithm, which led to a velocity-acceleration model that better matched real-world driving characteristics and significantly reduced errors by up to 23%. An innovative data-driven driving cycle development method based on minimum maximum ant colony optimization (MMACO) and the MC method was introduced to improve the representativeness of driving cycles in Ref.^20^, potentially serving as a benchmark for establishing fuel consumption standards. Gong et al.^21^ collected high-frequency operational data from battery electric vehicles (BEVs) and established the Beijing driving cycle through statistical and MC methods, laying a robust foundation for the precise evaluation of BEV performance in Beijing. The inaugural driving cycle for gasoline-powered vehicles was produced for the Greater Cairo of Egypt based on a diverse collection of high-resolution on-board measurements in Ref.^22^, which was superior in estimating fuel consumption and emissions.

In addition, many studies have delved into examining vehicle energy consumption and emissions by utilizing autonomously constructed actual driving cycles. Achour et al.^23^ estimated the contribution of private cars to local emission inventories based on a proposed representative driving cycle, and the strong representativeness of the driving cycle was verified by comparing with empirical measurements. The actual data from electric vehicles over six months were used to derive a driving cycle specifically tailored for evaluation in Ref.^24^, and energy consumption calculations indicated that the driving cycle adeptly mirrors the genuine local driving conditions. Ho et al.^25^ compared emissions of the Singapore Driving Cycle (SDC) and New European Driving Cycle (NEDC) using micro-estimation models, which revealed that NEDC underestimated most of vehicle emissions and SDC was more appropriate in Singapore. The online energy management of the Plug-in Hybrid Electric Vehicles (PHEV) was implemented using the dynamic programming (DP) algorithm based on the constructed actual driving cycle in Ref.^26^, and simulation results demonstrated a minimum 19.83% improvement in fuel efficiency compared to the charge depletion and charge sustain (CDCS) control strategy. Koossalapeerom et al.^27^ developed the driving cycle of electric motorcycles and measured the energy consumption, affirming the faithful reflection of the constructed cycle for real driving conditions. The fuel economy for both conventional and autonomous vehicles was precisely predicted according to the customized driving cycle in Ref.^28^, further emphasizing the necessity of enhancing fuel economy estimates through the use of customized driving cycles. Ma et al.^29^ introduced the AMarkov chain method to create representative driving cycles with actual driving characteristics, and the study underlined the significance of addressing real-world characteristics when improving fuel economy regulations. The CO_2_ emissions of five passenger cars were simulated in actual driving cycles in Ref.^30^, which revealed that local driving cycles were 30% closer to empirical data compared to the World Light-duty Vehicle Test Cycle (WLTC).

The constructed driving cycles exhibit diverse characteristics due to the varied objectives and methods adopted by researchers^31,32^. However, there is a lack of a complete systematic construction method from the collected original data to the actual urban driving cycles, thus improving efficiency, saving costs and facilitating comparisons. At the same time, the representativeness of the actual driving cycles constructed at present needs to be further improved, so as to reflect local driving characteristics more realistically. Currently, artificial intelligence (AI) has become increasingly ubiquitous across diverse domains, playing a pivotal role in numerous applications^33,34^. Deep learning is one of the essential components in the field of artificial intelligence^35^, which has become an important technology because of its exceptional generalization and prediction performance^36,37^. It is a novel and valuable research to construct driving cycles that are more relevant to the actual conditions by using deep learning as an effective tool. Therefore, a systematic driving cycle construction method called CS-DCC is proposed in this study, which integrates multi-scale wavelet analysis, KPCA, the Balanced Iterative Reducing and Clustering using Hierarchies (Birch) algorithm, and the improved autoencoder. The key contributions are delineated below.

- Electric vehicle road tests were conducted and relevant data were collected using the manual driving method.
- The CS-DCC method was proposed to systematically generate a representative driving cycle from the original data.
- The constructed driving cycle was compared with four standard driving cycles to verify the regional characteristics.
- The study introduced a new way to effectively use deep learning for constructing highly representative actual driving cycles.

## Methods

**Figure 1:**
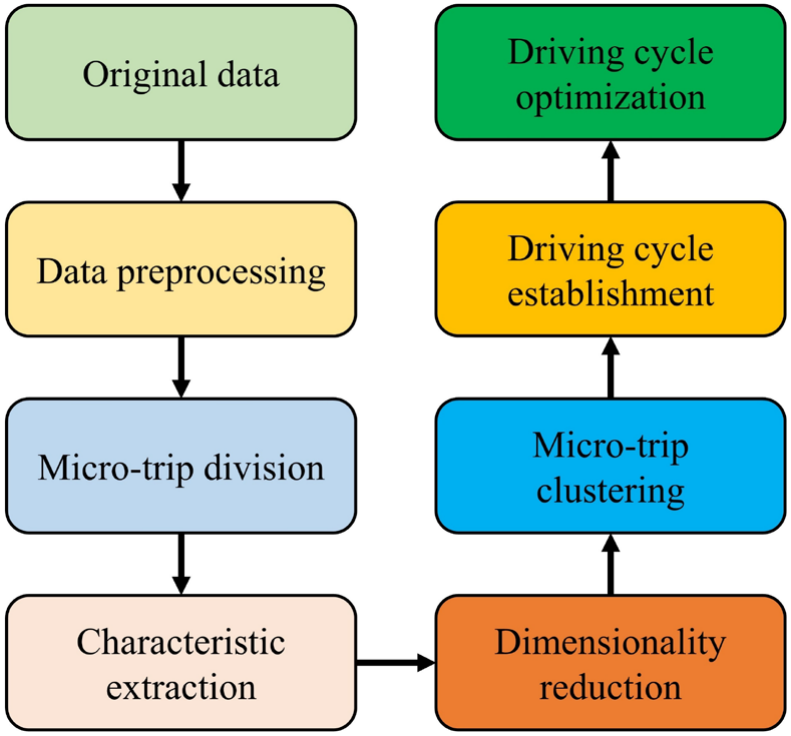
Workflow of the CS-DCC method.

In this study, the CS-DCC method is proposed to systematically generate a representative driving cycle from the original data, and the workflow is illustrated in Fig. 1. More specifically, the original data collected from electric vehicle road tests are sequentially subjected to seven steps, including data preprocessing, micro-trip division, characteristic extraction, dimensionality reduction, micro-trip clustering, driving cycle establishment and driving cycle optimization. These steps collectively lead to the creation of a highly representative actual driving cycle. During the data preprocessing phase, a sequential process is implemented involving missing data addressing, abnormal data handling and noise reduction, where multi-scale wavelet analysis is employed to mitigate data noise. In the micro-trip division step, the collected continuous data are segmented in a specific way according to the CS-DCC method. The characteristic extraction stage involves extracting characteristics from the micro-trips based on a predetermined set of 14 parameters specified in this algorithm. In the dimensionality reduction step, the KPCA algorithm is utilized to reduce the dimensionality of the characteristic matrix, which serves to alleviate computational complexity. The micro-trip clustering phase uses the Birch algorithm to cluster all micro-trips into three distinct classes based on predetermined criteria within the algorithm. During the driving cycle establishment stage, the Markov chain Monte Carlo (MCMC) method is applied to construct the driving cycle, leveraging the properties of stochastic processes with Markov characteristics. In the driving cycle optimization step, the constructed driving cycle is optimized based on the improved autoencoder to enhance its representativeness. It is worth noting that although the CS-DCC method is proposed based on city-specific road test data, it can still be applied to other road test data to generate actual driving cycles. More details about the seven steps are shown below.

**Figure 2:**
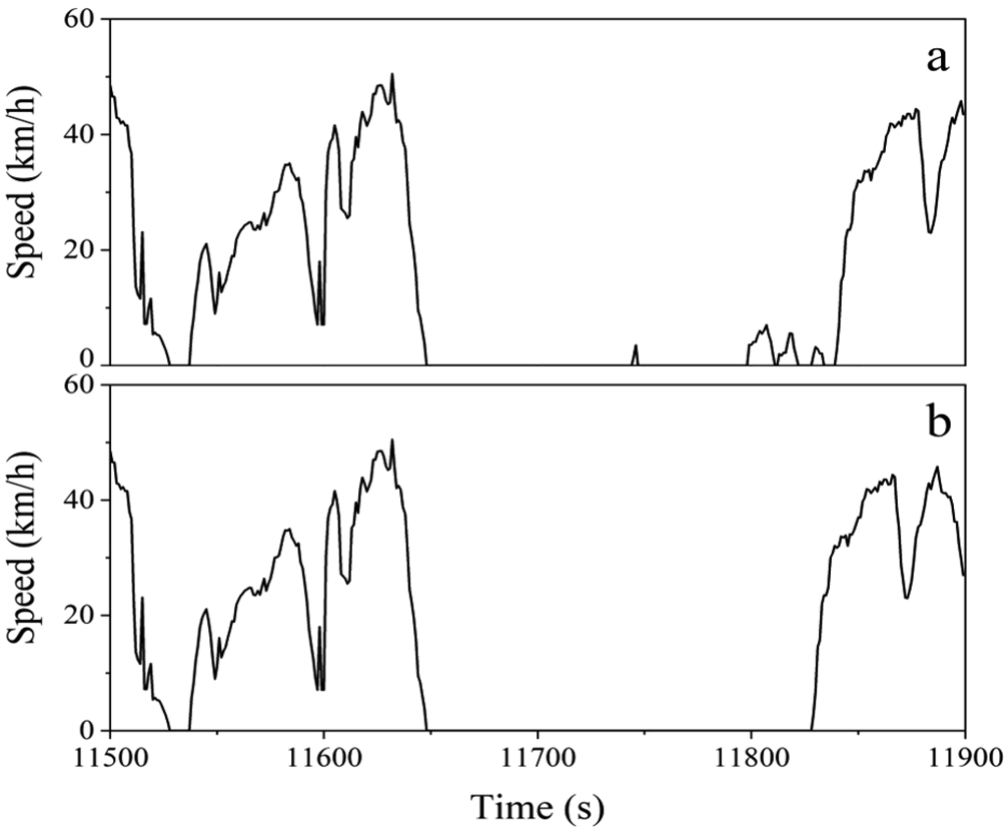
Speed-time curves before (**a**) and after (**b**) abnormal data processing.

The data preprocessing mainly includes two steps: (1) Interpolation of Missing and Abnormal Data; (2) Noise Reduction. The abnormal data are composed of driving at consistently low speeds intermittently and unusual acceleration patterns. Situations such as prolonged traffic congestion or intermittent low-speed driving (below 10 km/h within 30 s) are considered as an idling state, and the maximum continuous idling time is limited to 180 s. The processing method for intermittent low-speed driving is directly setting the value to 0 m/s and eliminating segments exceeding 180 s of idling, and the provided example in Fig. 2 demonstrates the comparison before and after this processing step. In this study, the time taken for the tested electric vehicles from 0 km/h to reach 100 km/h is assumed to be at most 7 s^38^, and the maximum deceleration during emergency braking is set at − 8 m/s^2^^39^. When an abnormal acceleration point is identified, the speed value is replaced by interpolation based on the surrounding 10 s data (5 s before and after), and then the acceleration at that point is adjusted accordingly. If abnormal acceleration persists even after processing, the timestamp of the data point is recorded and the micro-trip containing the abnormal data is removed. The driving behavior of a vehicle is a complex and random process, and the driving status is influenced by various factors, such as non-motorized vehicles driving on the motorway and diverse road conditions. As a result, there is a large amount of noise in the collected original data. To address this, a five-scale wavelet decomposition is employed for signal reconstruction through inverse transformation, effectively removing diverse noise sources. The micro-trip division entails segmenting the speed-time curve within a specific duration according to the trajectory of “end of idling—driving—end of idling”, which is a prevalent technique for segment division and can effectively realize the continuous combination of micro-trip.

Characteristic extraction involves the comprehensive characterization of the micro-trip by extracting essential parameters, and 14 parameters are selected in this study which are enumerated in Table 1 and calculated by Eqs. (1), (2), (3), (4), (5), (6), (7), (8), (9), (10), (11), (12), (13) and (14).

1 $$P_{a} = \frac{{T_{a} }}{T} \times 100\%$$

2 $$P_{d} = \frac{{T_{d} }}{T} \times 100\%$$

3 $$P_{i} = \frac{{T_{i} }}{T} \times 100\%$$

4 $$P_{u} = 1 - P_{a} - P_{d} - P_{i}$$

where *P*_*a*_, *P*_*d*_, *P*_*i*_ and *P*_*u*_ represent the time percentage corresponding to acceleration, deceleration, idling and uniform speed for the micro-trip, while *T*_*a*_, *T*_*d*_, *T*_*i*_ and *T*_*u*_ are the respective durations of these states; *T* is the total duration of the micro-trip.

5 $$v_{\max } = \max \left\{ {v_{i} ,i = 1,2, \cdots ,N} \right\}$$

6 $$v_{m} = \left( {\sum\limits_{i = 1}^{N} {v_{i} } } \right)/T$$

7 $$v_{md} = \left( {\sum\limits_{i = 1}^{N} {v_{i} } } \right)/\left( {T - T_{i} } \right)$$

8 $$v_{sd} = \sqrt {\frac{1}{N - 1}\sum\limits_{i = 1}^{N} {\left( {v_{i} - v_{m} } \right)^{2} } } ,{\kern 1pt} {\kern 1pt} {\kern 1pt} {\kern 1pt} {\kern 1pt} {\kern 1pt} {\kern 1pt} {\kern 1pt} i = 1,2, \cdots ,N$$

**Table 1:** 14 characteristic parameters selected from the basic evaluation criteria.

No	Parameter	Unit	Abbreviation
1	Time percentage of acceleration	–	*P*_*a*_
2	Time percentage of deceleration	–	*P*_*d*_
3	Time percentage of idling	–	*P*_*i*_
4	Time percentage of uniform speed	–	*P*_*u*_
5	Maximum speed	km/h	*v*_*max*_
6	Mean speed	km/h	*v*_*m*_
7	Mean driving speed	km/h	*v*_*md*_
8	Standard deviation of the speed	km/h	*v*_*sd*_
9	Maximum acceleration	m/s^2^	*a*_*max*_
10	Mean acceleration	m/s^2^	*a*_*am*_
11	Maximum deceleration	m/s^2^	*a*_*min*_
12	Mean deceleration	m/s^2^	*a*_*dm*_
13	Standard deviation of the acceleration	m/s^2^	*a*_*asd*_
14	Standard deviation of the deceleration	m/s^2^	*a*_*dsd*_

where *v*_*max*_, *v*_*m*_, *v*_*md*_ and *v*_*i*_ are the maximum, mean, mean driving and idling speeds, respectively; *v*_*sd*_ and *N* are the standard deviation of speed and total time, respectively.

9 $$a_{\max } = \max \left\{ {a_{i} ,i = 1,2, \cdots ,N - 1} \right\}$$

10 $$a_{am} = \frac{{\sum {a_{i}^{a} } }}{{T_{a} }},{\kern 1pt} {\kern 1pt} {\kern 1pt} {\kern 1pt} {\kern 1pt} {\kern 1pt} {\kern 1pt} i = 1,2, \cdots ,N - 1$$

11 $$a_{\min } = \min \left\{ {a_{i} ,i = 1,2, \cdots ,N - 1} \right\}$$

12 $$a_{dm} = \frac{{\sum {a_{i}^{d} } }}{{T_{d} }},{\kern 1pt} {\kern 1pt} {\kern 1pt} {\kern 1pt} {\kern 1pt} {\kern 1pt} {\kern 1pt} i = 1,2, \cdots ,N - 1$$

13 $$a_{asd} = \sqrt {\frac{1}{{N_{1} - 1}}\sum\limits_{i = 1}^{{N_{1} }} {\left( {a_{i}^{a} - a_{am} } \right)^{2} } } ,{\kern 1pt} {\kern 1pt} {\kern 1pt} {\kern 1pt} {\kern 1pt} {\kern 1pt} {\kern 1pt} {\kern 1pt} i = 1,2, \cdots ,N_{1}$$

14 $$a_{dsd} = \sqrt {\frac{1}{{N_{2} - 1}}\sum\limits_{i = 1}^{{N_{2} }} {\left( {a_{i}^{d} - a_{dm} } \right)^{2} } } ,{\kern 1pt} {\kern 1pt} {\kern 1pt} {\kern 1pt} {\kern 1pt} {\kern 1pt} {\kern 1pt} {\kern 1pt} i = 1,2, \cdots ,N_{2}$$

where *a*_*max*_ and *a*_*am*_ are the maximum and mean accelerations, respectively; *a*_*min*_ and *a*_*dm*_ are the maximum and mean decelerations, respectively; *a*_*asd*_ and *a*_*dsd*_ are the standard deviation of acceleration and deceleration, respectively; $a_{i}^{a}$ and $a_{i}^{d}$ are the acceleration and deceleration at the i-th second, respectively; *N*_1_ and *N*_2_ are the total time of acceleration and deceleration processes, respectively.

The 14 characteristic parameters extracted from micro-trips contain information pertaining to speed, acceleration and time percentage, which has overlapping and redundant information. To address this, the KPCA algorithm^40^ is employed in this study for dimensionality reduction to reduce computational complexity and improve efficiency. The KPCA algorithm operates on the kernel trick, enabling the kernelization of linear dimensionality reduction techniques to identify an appropriate low-dimensional embedding. This approach effectively mitigates dimensional discrepancies and preserves distinct information across each characteristic factor, ultimately enhancing the realism and reliability of the final outcomes.

In accordance with the classification criteria observed in the WLTC and China Light-duty Vehicle Test Cycle (CLTC), the vehicle speed is categorized into three classes which are low, medium and high speeds. In this study, all micro-trips are clustered using the Birch algorithm^41^ following these principles. The Birch algorithm is a robust and classical hierarchical clustering technique that can efficiently cluster data with a single scan and handle outliers, making it well-suited for managing extensive datasets. This distance-based hierarchical clustering method initially performs a bottom-up hierarchical coalescing process and subsequently employs iterative relocation to refine the results. During the hierarchical coalescing phase, individual objects are considered as atomic clusters and progressively combined to create larger clusters until all objects belong to a cluster or a specified end condition is met.

The process of establishing a driving cycle involves selecting specific micro-trips in a manner that constructs a speed-time curve of a defined length, endeavoring to capture the actual driving characteristics to the greatest extent possible. The MCMC method^42^ is employed for constructing the actual driving cycle in this study, which estimates the posterior distribution of interest parameters by utilizing random sampling in the probability space. Each subsequent sample in this process relies on the previous sample, thereby creating a stochastic process model with Markov properties. The Markov chain principle assumes that the probability of transitioning from one state to another at any given moment depends solely on the preceding state. This might seem arbitrary, but it serves as an effective approach to streamline the complexity of the model, substantially simplifying calculations. Mathematically, assuming that the state sequence is $\cdots X_{t - 2} ,X_{t - 1} ,X_{t} ,X_{t + 1} \cdots$, then the conditional probability of the state *X*_*t*+1_ depends only on the previous state *X*_*t*_:

15 $$P\left( {X_{t + 1} \left| { \cdots X_{t - 2} ,X_{t - 1} ,X_{t} } \right.} \right) = P\left( {X_{t + 1} \left| {X_{t} } \right.} \right)$$

The process of constructing the actual driving cycle through the MCMC method involves several steps: (1) Calculating the Markov chain state transition matrix P derived from the clustering results; (2) Given an arbitrarily set initial state distribution probability Z, obtaining the final stable probability distribution Q through the iterative action of Z and P; (3) Employing the Monte Carlo sampling technique to generate the driving cycle that satisfies specified conditions based on the derived probability distribution Q.

In order to create a driving cycle that authentically reflects the data characteristics of the vehicle under actual driving conditions, it is crucial not only to align with the real data distribution but also to minimize the average characteristic error with the entire dataset. Therefore, an improved version of the autoencoder^43^ is employed to optimize the constructed actual driving cycle, which learns the effective encoding of a dataset in an unsupervised way. The traditional autoencoder constructs a reconstruction loss based on the input and output data, which minimizes the gap between the output and input data by reducing the reconstruction loss, aiming to closely align the output with the input data. In this study, the constraints of the specific physical problem are integrated into the design of the autoencoder. Specifically, the average error of the characteristic parameters between the constructed driving cycle and the total data is taken as the characteristic loss, and a new loss is derived by weighted summation of the characteristic loss and the reconstruction loss. In this way, the driving cycle output from the improved autoencoder can maintain the essential driving characteristics of the original driving cycle under the constraint of the reconstruction loss. Meanwhile, the output driving cycle can further reduce the average error of the characteristic parameters with the total data under the constraint of feature loss, so as to improve the overall representativeness of the driving cycle.

16 $$L = \lambda_{1} L_{r} + \lambda_{2} L_{P}$$

17 $$L_{r} = \sum\limits_{t = 1}^{T} {\left( {x_{t} - x_{t}{\prime} } \right)}^{2}$$

18 $$L_{f} = \frac{1}{M}\sum\limits_{i = 1}^{M} {\left( {F_{i}{\prime} - F_{i}^{data} } \right)^{2} }$$

where $X = x_{1} ,x_{2} , \cdots ,x_{T}$ is the initial driving cycle, *x*_*t*_ is the vehicle speed at time *t*, *T* is the total cycle time, *Fdata i* represents the value of the i-th characteristic parameter of the total data, *M* is the total number of characteristic parameters, *L*_*r*_ and *L*_*f*_ are the reconstruction loss and the characteristic loss, respectively.

**Figure 3:**
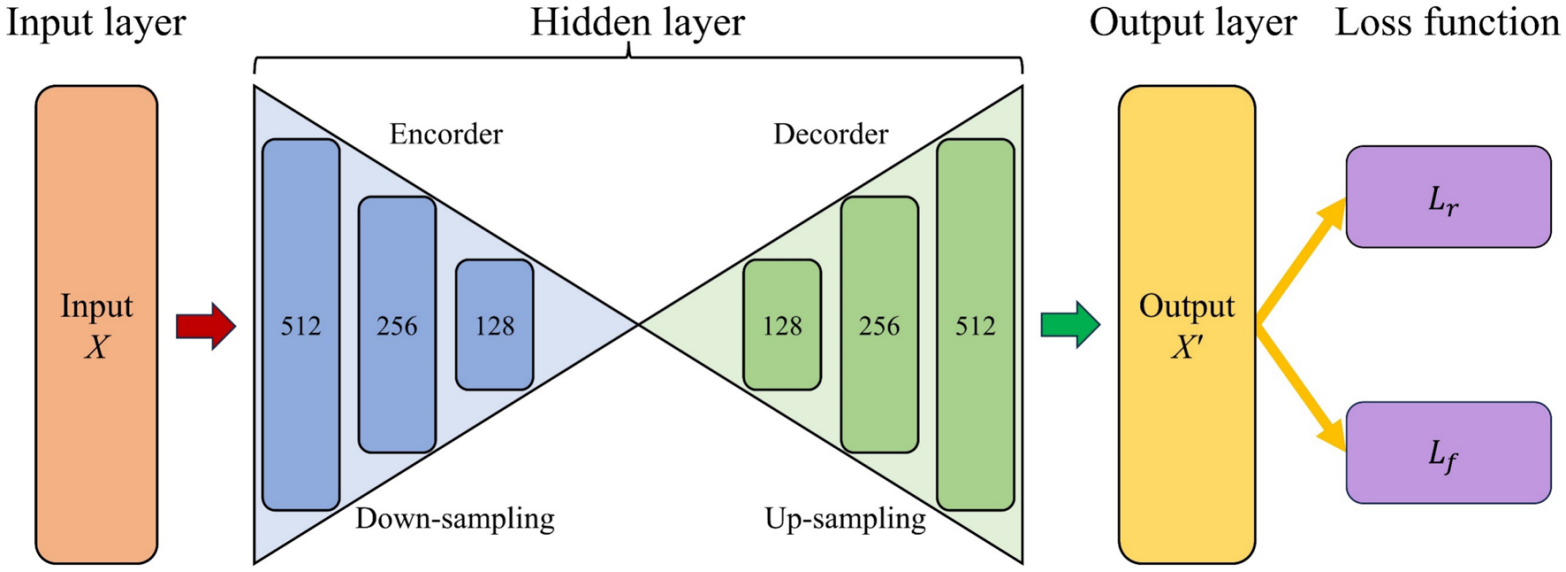
Structure of the improved autoencoder.

The structure of the improved autoencoder is presented in Fig. 3, which consists of three primary parts: the input layer, the hidden layer and the output layer. The driving cycle constructed based on the MCMC method is fed into the input layer, while the driving cycle optimized by the improved autoencoder is output from the output layer. The hidden layer comprises an encoder and a decoder, the encoder involves three fully-connected neural networks with a decreasing number of neurons for the purpose of down-sampling. Conversely, the decoder is comprised of three fully connected neural networks with an increasing number of neurons to facilitate up-sampling.

## Experiments and data

**Figure 4:**
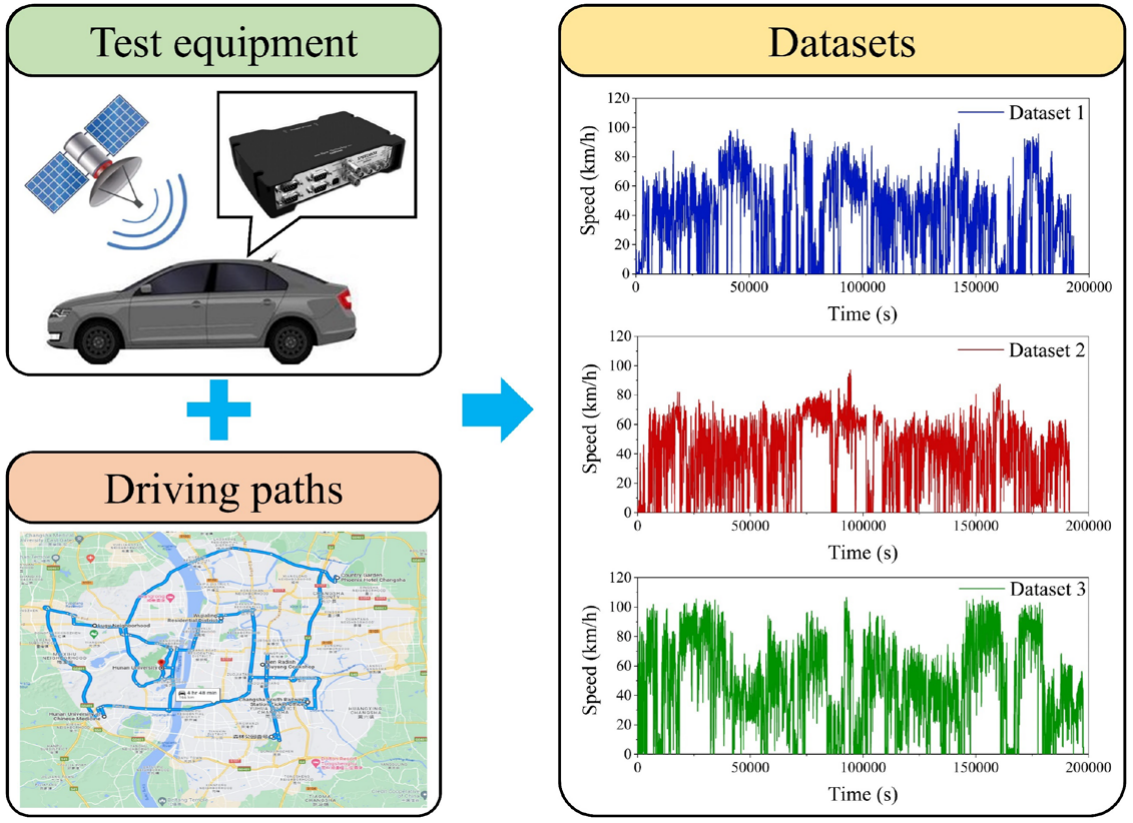
Test equipment, driving paths and datasets (Google Maps 2023, https://www.google.com/maps).

The experiment was conducted using three light-duty battery electric vehicles in Changsha, China. The data acquisition equipment comprised the Speed BOX data acquisition instrument, CAN module and transmission line, with a data acquisition frequency of 1 Hz. The Speed BOX serves as an input–output terminal capable of real-time signal collection, including vehicle speed, altitude, latitude, and longitude. Furthermore, it communicates with external systems through the CAN module, allowing reception of external signals such as accelerator and brake pedal opening signals. The primary signals recorded during vehicle operation include vehicle speed, torque and accelerator pedal opening. The manual driving method was employed to obtain the speed-time curve during actual driving, which granted drivers the freedom to conduct tests based on their driving habits without limitations concerning time, space or location. Consequently, the test data obtained through this manual driving method demonstrated a higher degree of randomness and closer approximation to real-world conditions. At the same time, a different driver was designated each day to prevent data collection from being influenced by driving styles. Considering fewer vehicles on the road during night and early morning hours, data collection was set between 7:00 a.m. and 6:00 p.m. daily. The test equipment, driving paths and datasets are illustrated in Fig. 4. Although only one driving path is displayed due to the multitude of paths, it is evident that the driving routes cover the city comprehensively.

## Results and discussions

**Figure 5:**
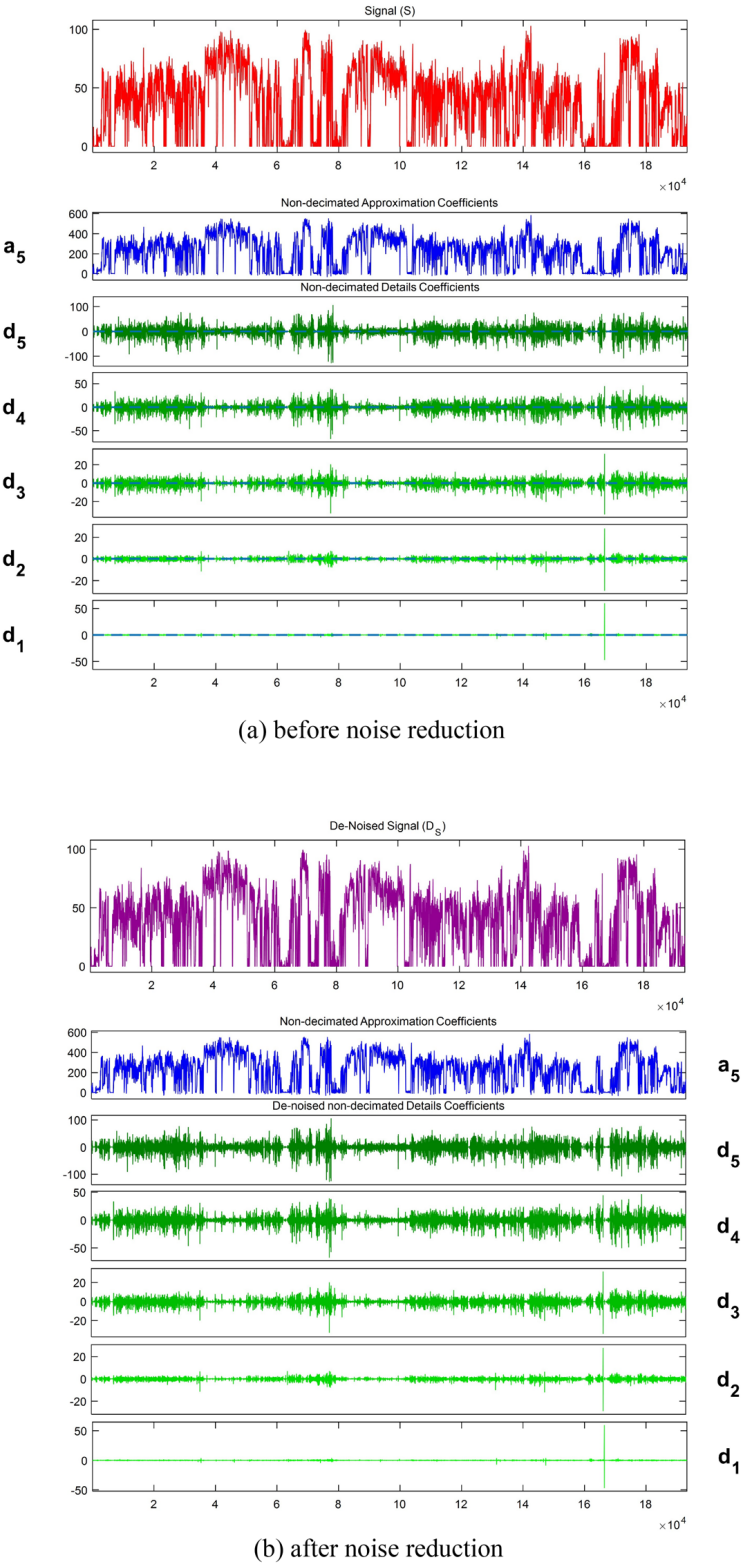
The first dataset with its approximation and detail coefficients before and after noise reduction.

**Figure 6:**
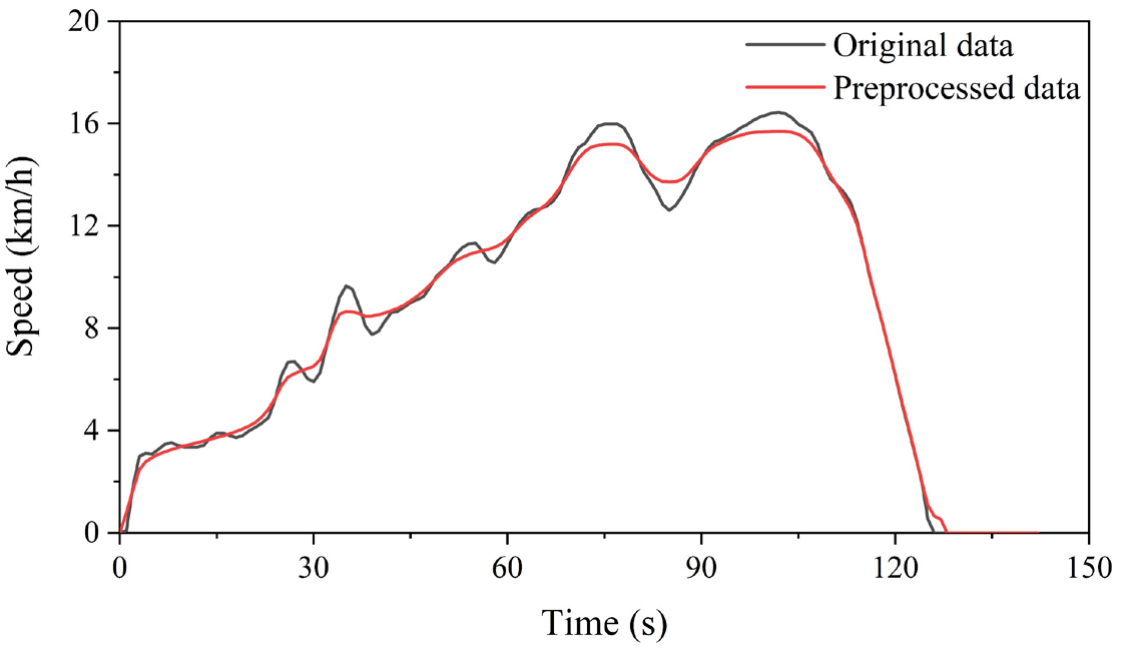
Change in a micro-trip within the first dataset before and after noise reduction.

Firstly, a total of 136 data points with missing information were identified after detailed examination, primarily resulting from weakened GPS signals when passing through tunnels or underground passages. In addition, 1115 abnormal data instances were initially detected, and this number was reduced to 295 through interpolation, which were found to be within 216 micro-trips. Consequently, these 216 micro-trips were excluded, resulting in 1058, 699 and 793 micro-trips extracted from the three datasets, summing up to a total of 2550 micro-trips as defined. The first dataset along with its approximation and detail coefficients under five-scale wavelet decomposition before and after noise reduction are illustrated in Fig. 5. The original data were subjected to five-scale wavelet decomposition, where the noise signal within the detail coefficients with higher frequencies was addressed using a gate threshold. Subsequently, the detail and approximate coefficients were wavelet reconstructed to achieve noise reduction. Considering the vast amount of data, the change in a micro-trip within the first dataset before and after noise reduction was specifically selected to illustrate the effect of multi-scale wavelet analysis, as shown in Fig. 6. It can be seen that the refined data after noise reduction exhibit greater stability and a smoother pattern in contrast to the original data.

**Figure 7:**
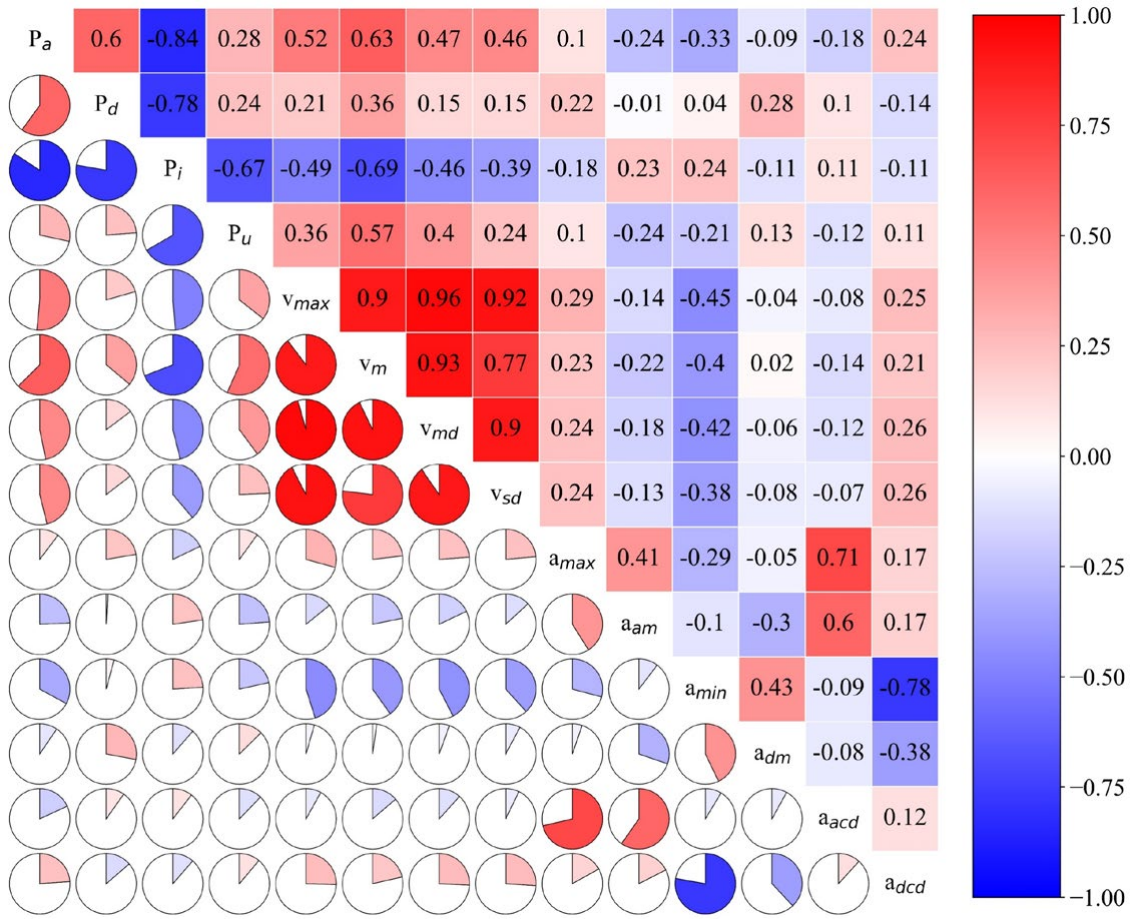
Pearson correlation coefficients between the 14 characteristic parameters.

**Figure 8:**
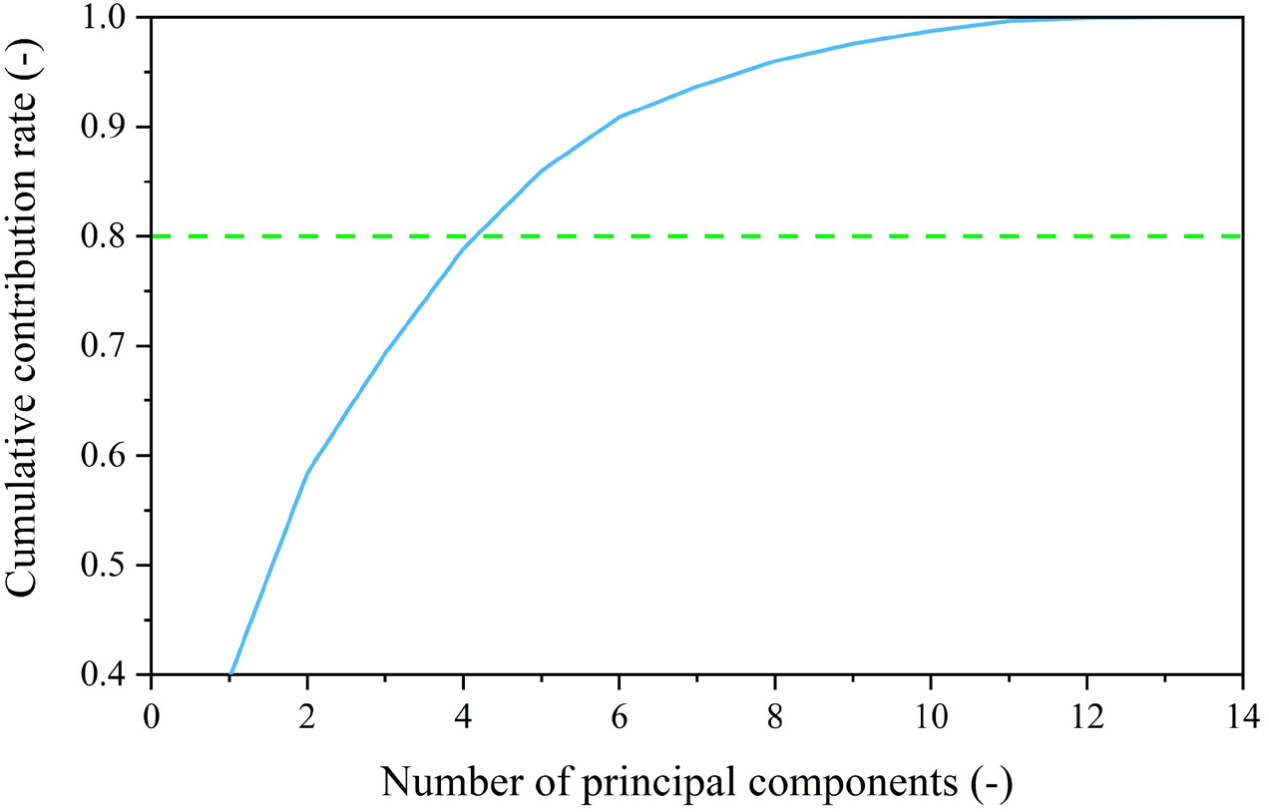
Variation in the cumulative contribution rate concerning the number of principal components.

Secondly, the Pearson correlation coefficients between the 14 characteristic parameters are demonstrated in Fig. 7 and calculated by Eq. (19):

19 $$Pearson_{XY} = \frac{{\sum\limits_{i = 1}^{n} {\left( {x_{i} - \overline{x}} \right)\left( {y_{i} - \overline{y}} \right)} }}{{\sqrt {\sum\limits_{i = 1}^{n} {\left( {x_{i} - \overline{x}} \right)^{2} } } \sqrt {\sum\limits_{i = 1}^{n} {\left( {y_{i} - \overline{y}} \right)^{2} } } }}$$

where $X = \left( {x_{1} ,x_{2} , \cdots ,x_{n} } \right)$ and $Y = \left( {y_{1} ,y_{2} , \cdots ,y_{n} } \right)$ are n-dimensional vectors; $\overline{x}$ and $\overline{y}$ are the mean values of *X* and *Y*, respectively. The Pearson correlation coefficient is an indicator to measure the linear correlation between two variables, which offers a straightforward calculation, easy comprehension, and widespread applicability. The value of the Pearson correlation coefficient ranges from − 1 to 1, where a value closer to 1 signifies a stronger positive correlation, while a value closer to − 1 indicates a stronger negative correlation. It can be seen that there are extremely strong positive or negative correlations among various variables, so it is necessary to reduce the dimensionality of the 14 characteristic parameters, thus avoiding unnecessary calculations and improving efficiency. Figure 8 presents the variation in the cumulative contribution rate concerning the number of principal components based on the KPCA algorithm. In this study, the criterion for selecting the number of principal components is based on a cumulative contribution rate of 80% and above. It can be observed that the cumulative contribution of the principal components stands at 85.99% when the number of principal components reaches 5. As a result, the determined number of principal components is 5. The principal component load matrix of the principal components and original characteristics is listed in Table 2, in which $r_{6,1} = 0.9467$ indicates that the original characteristic *X*_6_ has a strong correlation with the first principal component *Z*_1_. The principal component load matrix not only illustrates the robust representativeness of the five chosen principal components, but also emphasizes the validity and soundness of this selection.

**Table 2:** Principal component load matrix of the principal components and original characteristics.

Original characteristic	Principal component				
	*Z*_1_	*Z*_2_	*Z*_3_	*Z*_4_	*Z*_5_
*X*_1_	0.7926	− 0.0879	0.2613	0.3203	0.2776
*X*_2_	0.5678	− 0.3258	0.6598	0.0575	0.1894
*X*_3_	− 0.8072	0.2239	− 0.4710	− 0.2630	− 0.0428
*X*_4_	0.4497	− 0.1201	0.1445	0.2228	− 0.6732
*X*_5_	0.8887	0.2209	− 0.2231	− 0.2746	0.0482
*X*_6_	0.9467	0.0836	− 0.0315	− 0.1354	0.0401
*X*_7_	0.8789	0.2115	− 0.2562	− 0.2784	0.0514
*X*_8_	0.7985	0.2480	− 0.2935	− 0.3051	0.1126
*X*_9_	0.1395	0.6042	0.4589	− 0.4580	− 0.2039
*X*_10_	− 0.4708	0.5813	0.3206	− 0.0226	0.2675
*X*_11_	− 0.4353	− 0.6418	0.1194	− 0.4106	0.1439
*X*_12_	0.2218	− 0.6453	0.0349	− 0.3172	− 0.4296
*X*_13_	− 0.3877	0.5730	0.4745	− 0.3136	− 0.1896
*X*_14_	0.1620	0.6504	− 0.1486	0.5233	0.2251

**Figure 9:**
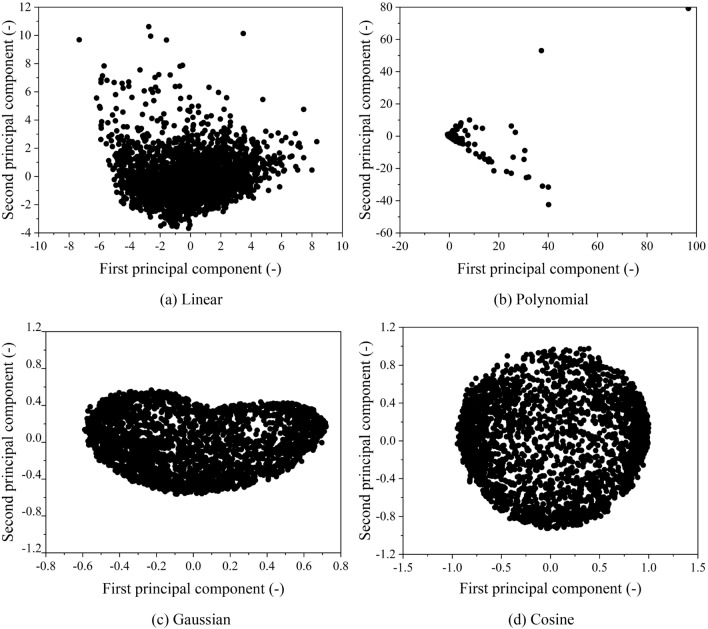
Scatter plots of the first and second principal components after dimensionality reduction by KPCA.

**Figure 10:**
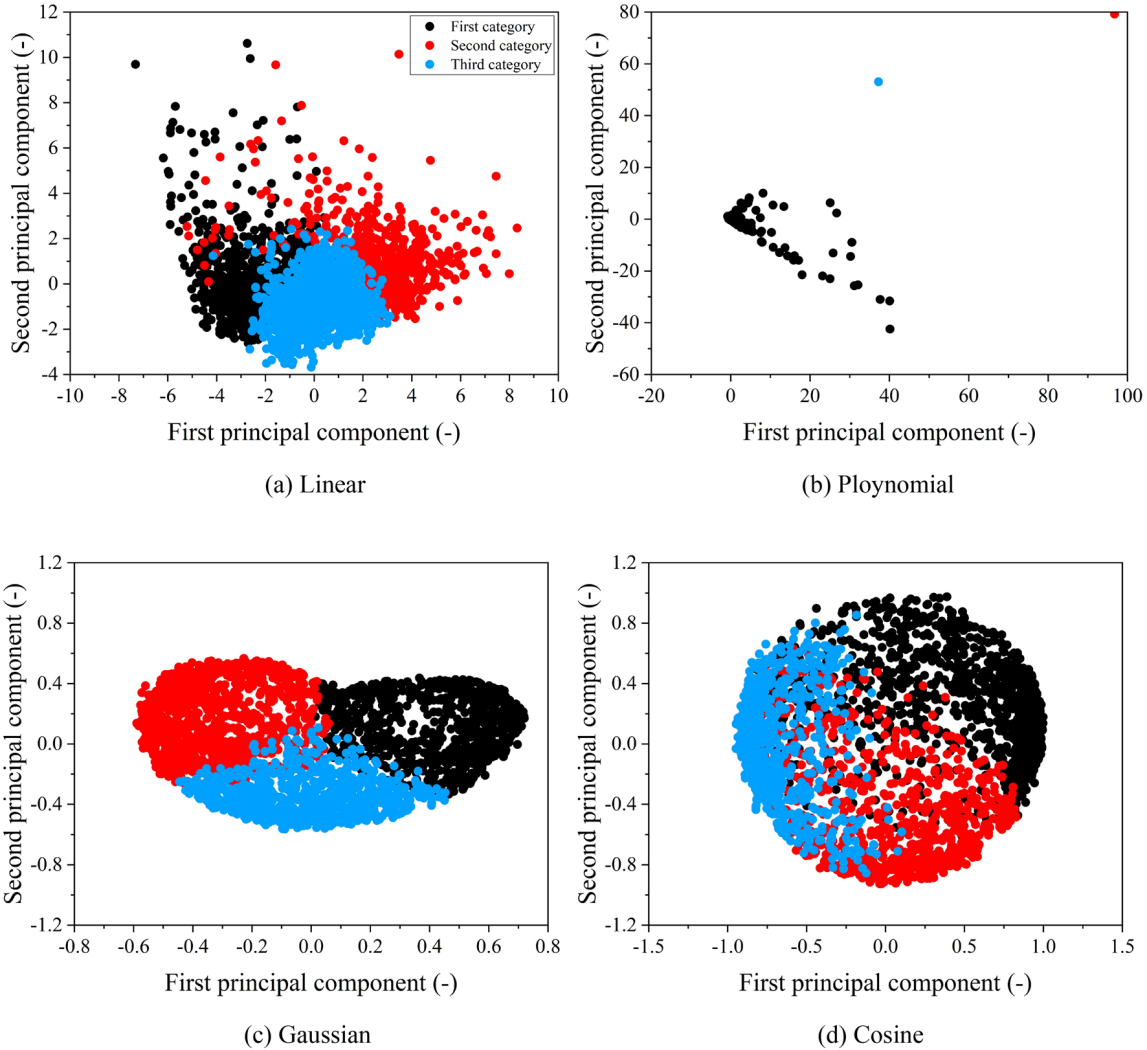
Scatter plots of the first and second principal components after clustering by Birch.

Thirdly, the KPCA algorithm using four distinct kernel functions was used to reduce the dimensionality of the matrix, which contained the characteristic parameters of all micro-trips. The comparison and analysis of these results were conducted to choose the optimal kernel function, considering that the number of principal components discussed earlier was determined to be five. The scatter plots of the first and second principal components after the dimensionality reduction based on the KPCA algorithm are displayed in Fig. 9, and it can be found that various kernel functions map the data into different high-dimensional spaces. Notably, the Gaussian KPCA and Cosine KPCA exhibit relatively dense data in high-dimensional spaces, while the Polynomial KPCA and Linear KPCA display scattered data. The micro-trips after dimensionality reduction by four KPCA algorithms were clustered using the Birch algorithm, and the results are given in Table 3 while the scatter plots of the first and second principal components are presented in Fig. 10. It should be noted that there is a significant difference in the number of three classes after dimensionality reduction by the Linear KPCA and Poly KPCA algorithms, which indicates that the results obtained using these two KPCA algorithms are notably less reasonable. Employing the Linear KPCA algorithm for dimensionality reduction results in 1725 micro-trips in the first class and only 214 in the third class, while using the Poly KPCA algorithm produces merely one micro-trip in the second class and 2537 micro-trips in the third class. On the contrary, the Gaussian KPCA and Cosine KPCA algorithms yield more balanced and coherent outcomes in terms of the distribution of micro-trips across the three classes, particularly the Gaussian KPCA algorithm. Considering the distribution of data in high-dimensional space and the class balance following clustering, the Gaussian KPCA algorithm is chosen for reducing the dimensionality of the characteristic matrix.

**Table 3:** Results of micro-trip clustering using the Birch algorithm.

Class	Linear	Poly	Gaussian	Cosine
1	1725	12	774	1203
2	611	1	972	934
3	214	2537	804	413

The characteristic parameters of the three classes obtained by the Gaussian KPCA and Birch algorithms are detailed in Table 4, which differ from each other. The first class exhibits a moderate speed, and the ratio of the acceleration and deceleration time is relatively high which may be in the unobstructed period on urban roads. The average speed of the second class is small and the proportion of idling time is high, suggesting probable scenarios of vehicle start-up or road congestion. The third class showcases high speed, a minimal proportion of idling time and a significant time percentage of acceleration, which may represent driving at high speeds in suburban areas.

**Table 4:** Characteristic parameters of three classes obtained by the Gaussian KPCA and Birch algorithms.

No	Parameter	Class 1	Class 2	Class 3
1	*P*_*a*_	0.522	0.350	0.377
2	*P*_*d*_	0.298	0.317	0.135
3	*P*_*i*_	0.145	0.322	0.094
4	*P*_*u*_	0.034	0.010	0.394
5	*v*_*max*_	72.4	36.5	101.5
6	*v*_*m*_	27.935	16.356	45.168
7	*v*_*md*_	30.807	21.118	56.465
8	*v*_*sd*_	17.449	14.831	34.170
9	*a*_*max*_	2.9	3.2	3.3
10	*a*_*am*_	0.379	0.524	0.264
11	*a*_*min*_	− 6.321	− 7.786	− 8.223
12	*a*_*dm*_	− 0.541	− 0.663	− 0.503
13	*a*_*asd*_	0.409	0.475	0.398
14	*a*_*dsd*_	0.595	0.864	0.541

**Figure 11:**
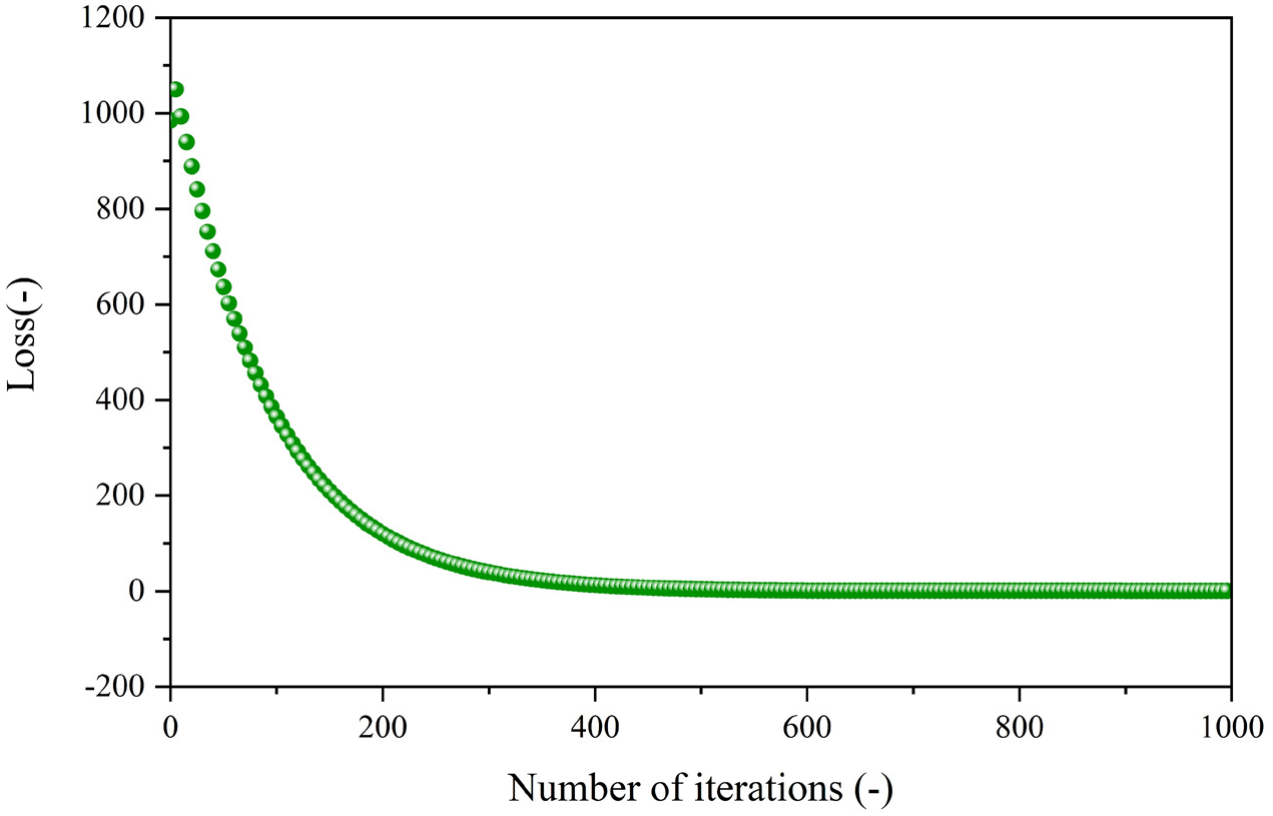
Loss function during the training process of the improved autoencoder model.

**Figure 12:**
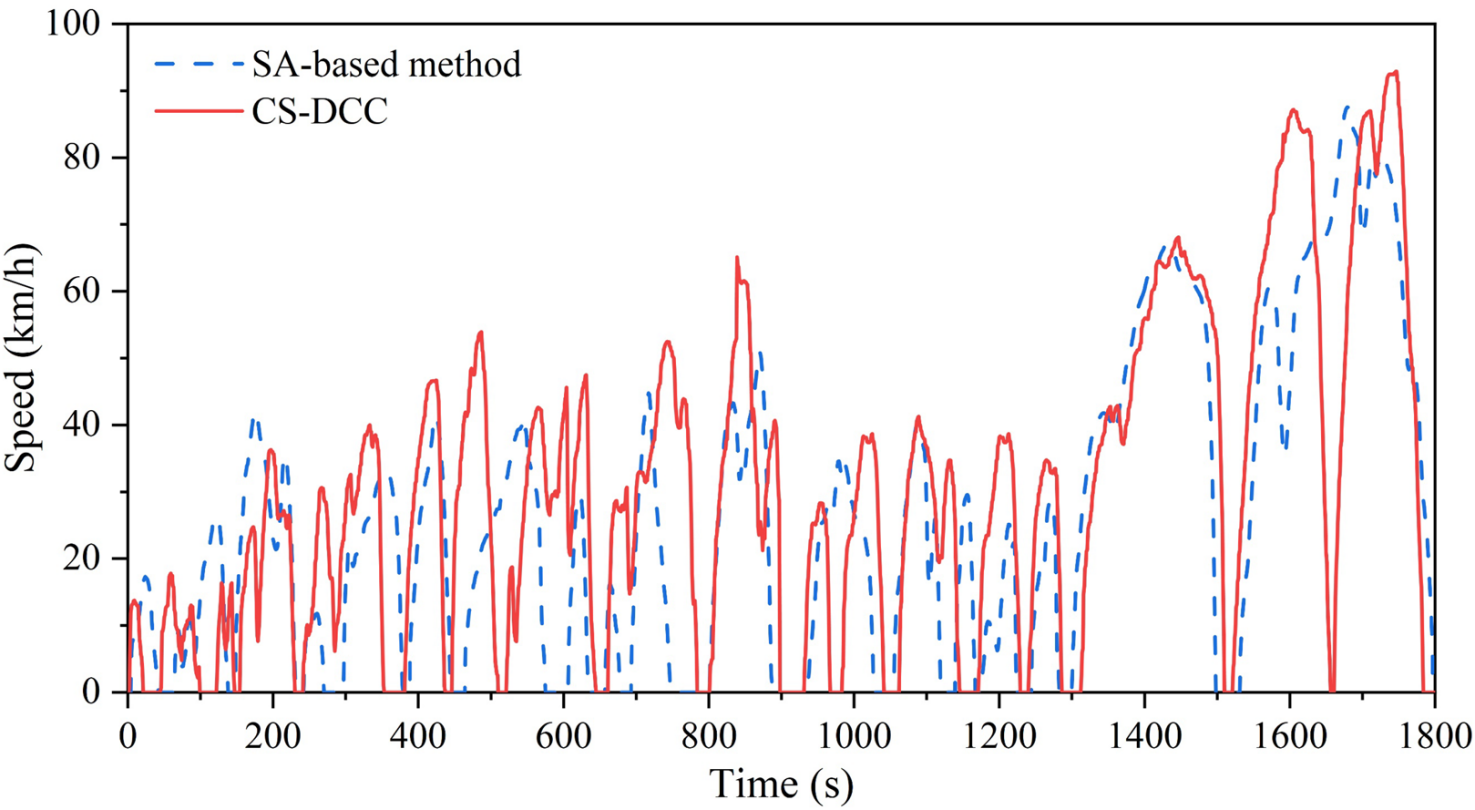
Driving cycles obtained based on the CS-DCC and SA-based methods.

Finally, the MCMC algorithm is applied to construct the actual driving cycle, which is further optimized by the improved autoencoder to enhance its representativeness. The loss function during the training process of the model is illustrated in Fig. 11, where the loss function gradually decreases and finally converges. The model undergoes training initially at a learning rate of 0.1 to accelerate the training for the first 30,000 iterations, followed by a learning rate adjustment to 0.01 to refine model accuracy after 30,000 iterations. The total training spans 50,000 iterations, resulting in a final loss of 0.9084, with loss outputs recorded every 50 iterations. The characteristic parameters of the constructed driving cycle before and after optimization compared with the total data are given in Table 5. The average error of the characteristic parameters between the optimized drive cycle and total data is notably reduced from 13.6% to 6.1%, with a reduction ratio of 55.1%. This reduction in error is achieved while preserving driving properties, showcasing the remarkable optimization performance of the improved autoencoder model. In order to demonstrate the effectiveness and advancement of the proposed CS-DCC method, a comparison is carried out based on the same dataset using the method developed in Ref.^19^, where a SA-based method is introduced. The driving cycles obtained based on the two methods are illustrated in Fig. 12. It can be seen that although the two methods use the same dataset, the constructed driving cycles consist of different micro-trips. The 14 characteristic parameters of the driving cycle obtained by the SA-based method are listed in Table 4, and the average error with respect to the total data is 9.7%. The characteristic parameters of the driving cycle obtained by the CS-DCC method have an average error of 6.1%, with an improvement ratio of 37.1%.

**Table 5:** Comparison between the 14 characteristic parameters of the constructed driving cycles and the total data.

No	Parameter	Total data	Before optimization	After optimization (CS-DCC method)	SA-based method^19^
1	*P*_*a*_	0.417	0.466	0.438	0.367
2	*P*_*d*_	0.250	0.315	0.274	0.243
3	*P*_*i*_	0.187	0.181	0.181	0.225
4	*P*_*u*_	0.146	0.038	0.107	0.164
5	*v*_*max*_	101.500	93.200	94.890	87.536
6	*v*_*m*_	29.820	30.419	30.407	26.107
7	*v*_*md*_	36.130	37.147	37.144	34.146
8	*v*_*sd*_	22.150	24.146	24.142	23.133
9	*a*_*max*_	3.263	3.389	3.367	2.980
10	*a*_*am*_	0.389	0.356	0.364	0.354
11	*a*_*min*_	− 8.223	− 7.778	− 7.978	− 7.266
12	*a*_*dm*_	− 0.569	− 0.525	− 0.582	− 0.493
13	*a*_*asd*_	0.427	0.354	0.412	0.399
14	*a*_*dsd*_	0.667	0.599	0.651	0.643
15	Average error	0	13.6%	6.1%	9.7%

The driving cycle constructed by the CS-DCC method is compared with four standard driving cycles and the results are shown in Table 6. It can be seen that the time percentage of acceleration in the constructed driving cycle exceeds that of the four driving cycles, while the ratio of idling time is lower than the Japanese 10–15 Mode (J10-15). However, the time proportion of uniform speed in the constructed driving cycle is the lowest. Notably, the average driving speed aligns closely with that of Urban Dynamometer Driving Schedule (UDDS), whereas the average acceleration is close to WLTC and significantly lower than the other three driving cycles. This comparison underscores the locally prominent characteristics present in the driving cycle generated through the CS-DCC method in contrast to standard driving cycles, which emphasizes the necessity of constructing an actual driving cycle that reflects localized driving patterns.

**Table 6:** Results of the constructed driving cycle compared with four standard driving cycles.

Cycle	*T*/(*s*)	*P*_*a*_/( −)	*P*_*i*_/( −)	*P*_*u*_/( −)	*V*_*m*_/(m·s^−1^)	*a*_*am*_/(m·s^−2^)
Constructed cycle	1800	0.4383	0.2744	0.1061	8.446	0.364
WLTC	1800	0.335	0.242	0.142	6.142	0.392
UDDS	1369	0.294	0.178	0.273	8.752	0.500
NEDC	1180	0.229	0.239	0.374	9.202	0.540
J10-15	660	0.261	0.326	0.195	6.300	0.540

## Conclusions

In this study, extensive efforts have been dedicated to the development of representative actual driving cycles. Electric vehicle road tests were conducted and relevant data were collected using the manual driving method, and the CS-DCC method was proposed to systematically generate a representative driving cycle from the original data. Besides, the constructed driving cycle was compared with four standard driving cycles to verify the regional characteristics, and the main conclusions are summarized as follows.

1. After noise reduction by five-scale wavelet analysis, the refined data exhibit greater stability and a smoother pattern in contrast to the original data. Analysis based on the Pearson correlation coefficients indicates the presence of extremely strong positive or negative correlations among the 14 extracted characteristic parameters.
2. Considering the distribution of the data in the high-dimensional space and the number of three classes after clustering, the Gaussian KPCA algorithm is chosen to reduce the dimensionality of the characteristic matrix. The number of principal components is determined as 5, and the cumulative contribution rate is 85.99%.
3. The characteristic parameters of the three classes obtained by the Gaussian KPCA and Birch algorithms differ from each other. The average error of the characteristic parameters between the optimized drive cycle and total data is notably reduced from 13.6 to 6.1%, with a reduction ratio of 55.1%, showcasing the remarkable optimization performance of the improved autoencoder model.
4. The proposed CS-DCC method demonstrates an effective method for constructing highly representative actual driving cycles, and the constructed driving cycle has obvious local characteristics in contrast to four standard driving cycles, which emphasizes the necessity of constructing an actual driving cycle that reflects localized driving patterns.

All of these not only provide an efficient method for the methodical construction of a representative driving cycle from original data, but also present a powerful application of artificial intelligence in advancing engineering technologies.

## Data Availability

The datasets used and/or analysed during the current study available from the corresponding author on reasonable request.

## Abbreviations

AI: Artificial intelligence
BEVs: Battery electric vehicles
Birch: Balanced iterative reducing and clustering using hierarchies
CDCS: Charge depletion and charge sustain
CLTC: China light-duty vehicle test cycle
CS-DCC: Changsha driving cycle construction
DP: Dynamic programming
J10-15: Japanese 10–15 mode
KPCA: Kernel principal component analysis
MC: Markov chain
MCMC: Markov chain Monte Carlo
MMACO: Minimum maximum ant colony optimization
NEDC: New European Driving Cycle
NN: Neural networks
PCA: Principal component analysis
PHEV: Plug-in hybrid electric vehicle
SA: Simulated annealing
SDC: Singapore driving cycle
SVM: Support vector machine
UDDS: Urban dynamometer driving schedule
WLTC: World light-duty vehicle test cycle
